# An Exploration of Practitioners’ Experiences of Delivering Digital Social Care Interventions to Children and Families During the COVID-19 Pandemic: Mixed Methods Study

**DOI:** 10.2196/43498

**Published:** 2023-04-28

**Authors:** Grainne Hickey, Claire Dunne, Lauren Maguire, Niamh McCarthy

**Affiliations:** 1 Barnardos Ireland Dublin Ireland; 2 Technological University Dublin Dublin Ireland

**Keywords:** digital social care, social care practice, children and families, COVID-19, mobile phone

## Abstract

**Background:**

Digital technology is an increasing feature of social care practice, and its use has accelerated greatly in response to the COVID-19 pandemic.

**Objective:**

This study aimed to assess social care practitioners’ experiences of delivering digital interventions to vulnerable children and families during the pandemic.

**Methods:**

A mixed methods study combining survey and qualitative research was conducted. In total, 102 social care practitioners working in the Republic of Ireland who delivered a range of digital social care support took part in a web-based survey. This survey captured practitioners’ engagement and experiences of delivering digital social care interventions to children and families as well as training and capacity building needs. Subsequently, 19 focus groups with 106 social care practitioners working with children and families were also conducted. These focus groups were directed by a topic guide and explored in more depth practitioners’ perceptions of digital social care practice, the perceived impact of digital technology on their work with children and families, and the future application of digital social care interventions.

**Results:**

The survey findings revealed that 52.9% (54/102) and 45.1% (46/102) of practitioners, respectively, felt “confident” and “comfortable” engaging in digital service delivery. The vast majority of practitioners (93/102, 91.2%) identified maintaining connection during the pandemic as a benefit of digital social care practice; approximately three-quarters of practitioners (74/102, 72.5%) felt that digital social care practice offered service users “increased access and flexibility”; however, a similar proportion of practitioners (70/102, 68.6%) identified inadequate home environments (eg, lack of privacy) during service provision as a barrier to digital social care practice. More than half of the practitioners (54/102, 52.9%) identified poor Wi-Fi or device access as a challenge to child and family engagement with digital social care. In total, 68.6% (70/102) of practitioners felt that they needed further training on the use of digital platforms for service delivery. Thematic analysis of qualitative (focus group) data revealed 3 overarching themes: perceived advantages and disadvantages for service users, practitioners’ challenges in working with children and families through digital technologies, and practitioners’ personal challenges and training needs.

**Conclusions:**

These findings shed light on practitioners’ experiences of delivering digital child and family social care services during the COVID-19 pandemic. Both benefits and challenges within the delivery of digital social care support as well as conflicting findings across the experiences of practitioners were identified. The implications of these findings for the development of therapeutic practitioner–service user relationships through digital practice as well as confidentiality and safeguarding are discussed. Training and support needs for the future implementation of digital social care interventions are also outlined.

## Introduction

### Background

Digital technology is an increasingly prominent feature of health care delivery; however, social care has remained less digitized than other systems [1]. As an allied health profession, child and family social care practice is aimed at supporting the welfare and safety of vulnerable children and families. Similar to many other health professionals, social care practitioners develop an understanding of clients’ needs and provide support through the development of interpersonal relationships [2]. Traditionally, this involves physical proximity and face-to-face interaction, often in the context of the home environment [3]. Digital technologies, however, have crept into the delivery of social care interventions and are increasingly being used to deliver programs and support to children and families [4,5].

Research has highlighted the potential opportunities afforded by digital social care work, including increased flexibility and accessibility as well as reduced costs and barriers to engagement [6,7]. Importantly, studies have also demonstrated that therapeutic support for children and families can be effectively adapted to digital platforms and can result in improvements in parent and child outcomes [8,9].

More generally, service users have been found to report high satisfaction with digital service delivery as well as positive relationships and strong alliance with service providers when working through these platforms [10,11]. Digital services may also be potentially beneficial for those who struggle to engage in face-to-face support and in instances where distance from the practitioner might strengthen their ability to participate in an intervention [12,13]. For instance, some clients may find it easier to communicate and discuss sensitive issues on the web [14]. In particular, young people may experience a greater sense of empowerment and feelings of control over their relationship with social care practitioners when communicating through digital technologies [12].

Despite this, previous studies have highlighted practitioners’ reluctance to engage in digital social care because of concerns that technological devices may limit their ability to observe and appropriately assess service users’ needs and safety [15]. Apprehensions around developing and sustaining therapeutic alliance through digital technologies have been reported [16]. For example, delays, dropouts, background noise, and other such technological difficulties may disrupt participant-practitioner engagement and relationship building. Inadequate digital skills among service users and practitioners may impede communication. Likewise, insufficient access to technological devices or appropriate spaces to speak openly with a professional social care worker may also be problematic, particularly for disadvantaged and vulnerable groups [17].

The implications of digital intervention for confidentiality within service user–practitioner relationships and interactions also require careful consideration. Research exploring the implementation of digital social care has highlighted how therapeutic sessions have been disturbed by the presence of others, including, in some instances, an abusive family member and practitioners or service users being recorded without consent or unknowingly observed or overheard by a third party [18,19]. Feelings of uncertainty or fear with regard to privacy may undermine trust and, in turn, the extent to which service users will reveal intimate information about their lives [20].

During the pandemic, social care practitioners rapidly pivoted to the use of digital technologies to provide support and ensure the protection of vulnerable children and families [21]. Emerging evidence during this period has highlighted the benefits of digital technology for social care work with children and families, such as enhanced responsivity to service users and increased engagement with and between service users [22,23]. However, this rapid swing toward digital social care was not without tension due to gaps in knowledge, guidelines, and infrastructure regarding how to safely and ethically deliver these types of interventions to vulnerable children and families [24].

Recent studies have shown that the pandemic has resulted in significant mental health burden in children and adolescents [25,26], and families from disadvantaged and vulnerable backgrounds have been disproportionately affected and have experienced greater rates of stress and anxiety [27,28]. Research has also suggested that the risk of child maltreatment was exacerbated during lockdowns [29-31]. It is likely that a greater number of families will require social care support. Increasingly, child and family social care practitioners will be required to engage with service users in new and innovative ways, including the use of digital and blended approaches. It is vital, therefore, that gaps in our understanding of digital social care practice are addressed to ensure that children and families receive effective support [32].

The overarching aim of this evaluation was to explore social care practitioners’ experiences of digital practice during the COVID-19 pandemic. The specific objectives were to (1) survey engagement with digital service delivery, (2) explore the experience of working remotely with children and parents through digital platforms in the context of the COVID-19 pandemic, and (3) capture digital social care practice training and support needs among practitioners working with vulnerable children and families.

### Study Context

This study was conducted in the Republic of Ireland as part of an internal evaluation of digital social care work with children and families delivered by Barnardos Ireland during the COVID-19 pandemic. Barnardos Ireland is a children’s charity and frontline service provider who works directly with children and families who are affected by adverse childhood experiences, such as emotional, physical, or sexual abuse; emotional or physical neglect; and family disruption such as separation, bereavement, domestic violence, criminality, and parent mental health difficulties [33]. Services provided by Barnardos include practical family support, evidence-based individual and group work programs for children and parents, one-to-one therapeutic support, early years and early intervention services, and specialist services such as bereavement and postadoption support. These services aim to promote child safety and well-being through interventions designed to enhance protective factors (eg, positive parenting, parent mental health, child resilience, and coping skills) and reduce risk factors (eg, parent substance abuse and domestic violence).

Staff at Barnardos Child and Family Services have a recognized third-level practice qualification (minimum level 7 on the Irish National Framework of Qualifications) in 1 of the following fields: social work, psychology, social care, early childhood care and education, and youth work. In total, there were 140 practitioners (known as project workers) working within the organization, 40% (56/140) of whom had between 1 and 5 years of experience in the service. A further 5% (7/140) of the project workers had between 5 and 10 years of service experience, 15.7% (22/140) of the project workers had ≥10 years of experience in the organization, and 16.4% (23/140) of the project workers had worked in the organization for <1 year.

During the COVID-19 pandemic, Barnardos responded to ensure that essential services could continue to be delivered to vulnerable parents and children across Ireland. A COVID-19 response plan was developed to outline the adaptation and innovations in delivering services. This included a “layered approach” to service delivery, which varied according to the presenting needs of the parent, adult, or child, with practitioners working to identify the most vulnerable families and developing holistic interventions in response. During this period, digital interventions included “check ins” via phone; video calls; web-based individual and group support programs with children, parents, and adult service users; and the development of a national parent support line and a well-being–focused web page of resources for parents and children. Face-to-face sessions in centers, doorstep drops of food and other essential supplies, and home visits in cases where there were concerns about children and their parents were also conducted. During lockdown periods, focus on safety planning, establishing, and maintaining routines; offering support to manage crises; and supporting engagement in homeschooling was prioritized. Implementation support for digital social care interventions included the provision of smartphones and laptops to Barnardos staff members. A number of devices were also made available to families to engage in Barnardos services and web-based learning during lockdown periods. A guide for digital working with families was developed for staff members working with children and families using digital platforms.

## Methods

### Study Design

A mixed methods exploration of practitioners’ experiences of digital social care practices during the COVID-19 pandemic was conducted. The research design and methodologies were developed by the Barnardos Ireland best practice team. Overall, 2 members of this team, both of whom were qualified social care practitioners, collected data between April and July 2021. The role of the best practice team is to provide support for the ongoing development and delivery of high-quality, evidence-based, needs-led, and outcome-focused services within Barnardos Ireland.

### Measures

A web-based survey was developed to explore staff members’ experiences of delivering digital social care interventions during the COVID-19 pandemic. This survey captured practitioners’ engagement and confidence in working digitally, perceptions of adapting programs and practices to digital platforms and perceived advantages and disadvantages of digital practice, as well as training and capacity building needs. Questions predominately consisted of multiple-choice format items (eg, During online work how often did you experience issues with your internet connection? “Very often,” “Often,” “Sometimes,” “Rarely,” and “Never”). A small number of multiple-response items were included in the survey, whereby respondents were permitted to provide >1 response (eg, In your experience, what have been the benefits for children and families of working online? From the list below, choose the options that have applied the most: “Maintaining connection during the pandemic,” “Increased access/flexibility for parents who are unable to attend due to work,” “Increased access for parents who cannot usually attend due to geographical barriers/travel requirements,” and “Improved access for participants who would never attend face-to-face service”). A small number of open questions were also included to allow respondents to provide a more detailed response (eg, “Please share any additional thoughts, ideas, or comments in relation to online service delivery”).

A total of 19 focus groups were also conducted with staff members from across the Barnardos organization. This qualitative data collection enabled more in-depth exploration of the views and preferences of children, families, and staff members in relation to digital service delivery. Focus group topic guides were devised, which consisted of 10 open-ended questions exploring practitioners’ experiences of working on the web with child and adult service users (eg, “How have you experienced working with new referrals/service users online?”), the perceived impact of digital technology on their practice (eg, “To what extent do you feel that you had to adapt your communication style or use different skills when working online?”), and the future application of digital social care interventions (eg, “What level/type of training would be useful?”).

### Participant Recruitment and Data Collection Procedures

Recruitment of practitioners for the research was conducted via email. All Barnardos staff members were sent an information sheet outlining the purpose of the survey and were asked to provide informed consent for data collection. A link to the web-based survey was provided using Microsoft Forms. The survey data were collected anonymously. The participants took on average 17-20 minutes to complete the survey.

Subsequently, 20 Barnardos project teams were purposively selected for participation in focus groups based on region, urban or rural location, and service type (eg, size, services provided, and communities supported) to ensure a representative spread of staff members from across the organization. Project team leaders were invited via email to participate in a separate, once-off focus group interview. A total of 19 project team leaders accepted the invitation to participate, and 1 project team leader declined the invitation because of time pressure. Staff members within the participating projects were provided with information sheets regarding the purpose and processes involved in the focus groups, and informed consent was obtained. A total of 106 Barnardos staff members participated in these consultations.

Focus groups, which were facilitated by 2 members of the Barnardos best practice team, were conducted on the web and recorded with consent. The focus groups lasted approximately 90 minutes. The recordings were subsequently transcribed using a nonverbatim approach. This approach focused on capturing the meaning inherent in speech extracts, rather than word for word reproduction. Colloquialisms, filler words or repetitions, and “off-topic” comments were excluded, but verbatim records of comments or speech were transcribed for key points made by participants.

### Ethics Approval

The study was approved by the Barnardos Services Review Committee. Information sheets outlining the purpose and nature of the research were circulated before data collection, and written informed consent was obtained from all participants. The survey data were collected anonymously. All focus group participants were assured of confidentiality, and qualitative data were deidentified following transcription.

### Data Analysis

Quantitative analyses included appropriate descriptive statistics. Responses to the open-ended survey items were subjected to content analysis. The focus group data were analyzed using a thematic analysis approach, which involved 4 key stages: familiarization, coding, defining themes, and interpretation. This process was supported using NVivo 11 (QSR International), a qualitative data analysis software package. The familiarization stage involved in-depth reading of all qualitative data and generating detailed summaries of the focus group transcripts. Subsequently, initial codes to explain the data were generated to capture the meaning of the data, which led to the specification of perspectives and experiences inherent in the data. The later stages of analysis involved integrating codes into overarching themes. Finally, the conclusions and interpretations were assessed to determine the strength and depth of the findings or themes. For example, the prevalence of perspectives was examined, and findings from different data sources (survey and focus groups) were also compared. This process of data triangulation was used to develop a full perspective of our findings and conclusions.

## Results

### Participants

A total of 102 Barnardos staff members completed the anonymous web-based survey. This included staff delivering family support (58/192, 56.8%), specialized services (eg, postadoption services; 11/102, 10.7%), and universal services (eg, group-based community interventions; 8/102, 7.8%). Survey participants included 49 practitioners working in the south and southeast of the country (49/102, 48%), and the remainder were located in Dublin North (25/102, 24.5%), Dublin South (10/102, 9.8%), and Dublin City Centre and Midlands regions (17/102, 16.6%).

A total of 106 Staff members took part in the focus groups. Services and supports delivered by focus group participants on a routine basis included early years interventions and supports for young children; intensive parent, child, and family work; and universal parent and child programs.

### Survey Findings

#### Practitioners’ Experiences of Delivering Digital Social Care Services

Participating staff members predominantly used videoconferencing (eg, Zoom [Zoom Video Communications]) to deliver services via the web (86/102, 84.3%), WhatsApp video calls (54/102, 52.9%), and standard phone calls. Most participants (89/102, 87.2%) felt that they were able to work well on the web. Survey responses also indicated that working on the web was welcomed by a significant proportion of participants (68/102, 66.7%) as “a chance to be creative and try new ways of working.” Respondents were provided with a list of feelings and asked to select the words that best described how they felt about working on the web. “Confident” (54/102, 52.9%) and “comfortable” (46/102, 45.1%) were the most frequently selected; 21.6% (22/106) of the respondents selected “safe” as a descriptor of how they feel when working on the web. However, just over one-quarter of the participants (28/102, 27.5%) selected “frustrated.” The words “unsafe” or “uneasy” were selected by a small proportion of respondents (3/102, 2.9% and 8/102, 7.8%, respectively). The word “Neutral” was selected by 22.5% (23/102) of respondents, whereas 16.7% (17/102) of respondents selected “other” and provided further details to explain their response. These details that revealed staff members’ sense of anxiety when experiencing connection issues as well as concerns about the ability to accurately assess child safety and difficulty in reading nonverbal cues, providing emotional support, and demonstrating empathy for service users during sensitive conversations. Staff members were also asked, “Do you feel that the skills you use to work with children and families are transferrable to online work?” Almost all staff members (86/102, 84.3%) responded “yes” to this question, and a small number of staff members (13/102, 13%) selected a “no” response.

#### Perceived Benefits and Challenges of Digital Social Care for Children and Families

The survey explored the perceived benefits and challenges of digital social care for children and families using 2 multiple-response items. When asked “what have been the benefits for children and families of working online,” the vast majority of respondents (93/102, 91.2%) identified “maintaining connection during the pandemic” as a benefit, approximately three-quarters of respondents (74/102, 72.5%) agreed that digital social care practice offered “increased access/flexibility for parents who are unable to attend due to work,” and just more than half of the respondents (51/102, 50.1%) felt that “increased access for parents who cannot usually attend due to travel requirements” was a benefit of digital practice. A smaller proportion of respondents (38/102, 37.3%) felt that the benefit of digital social care was “improved access for participants who would never attend face-to-face services.”

When asked “what are the barriers/challenges for children and families using digital services,” the following responses were recorded: 68.6% (70/102) of respondents identified inadequate home environments during service provision (eg, because of a lack of childcare and privacy), just more than half of survey respondents (56/102, 54.9%) identified “poor connection or limited access to devices/Wi-Fi” as a barrier to children’s and families’ engagement with digital social care, and 52.9% (54/102) of survey respondents identified service user capacity as an engagement barrier. In total, 50.9% (52/102) of respondents identified a lack of access to appropriate devices as a barrier to service user engagement in digital social care interventions.

#### Training Needs

More than three-quarters of the survey respondents (70/102, 78.6%) felt that they needed further training in the use of web-based or digital platforms for service delivery; more than half of the survey participants (56/102, 54.9%) felt that they needed greater practical advice on using digital tools. In addition, 38.2% (39/102) of participants perceived a need for training on how to create safety on the web for participants as well as guidance on mandatory tasks such as obtaining consent and impact measurement.

### Qualitative Findings

The qualitative findings were divided into 3 overarching themes and a range of subthemes (Textbox 1).

**Overview of themes from focus groups with service providers.**

- Perceived advantages and disadvantages of digital social care
  Perceived advantages for service users
  Perceived disadvantages for service users
- Practitioner challenges in delivering digital social care interventions
  Developing and sustaining relationships on the web
  Confidentiality and safeguarding
  Assessing the suitability of web-based service delivery
- Practitioners’ personal challenges and training needs

### Perceived Advantages and Disadvantages of Digital Social Care

#### Perceived Advantages for Service Users

Focus groups with staff members highlighted flexibility as a significant benefit of digital services for families. Digital social care services were seen as helping to accommodate parents who were in full-time employment as well as those who usually experienced childcare and practical barriers (eg, travel) to attending face-to-face, center-based services:

Parents don’t need to arrange childcare to attend a service now or if a child was sick at home, the parent doesn’t need to cancel or delay the programme.

When you think of it, it’s very difficult for a parent who works full-time to access our services...of course we accommodate families as much as we can, we always do but often those families end up closing or cant engage in a meaningful way.

...with teenagers it can work well as they usually come to the service after school, it can be a bit rushed to get home, have their dinner and get down to the project, but with online working they can log straight on after school, they don’t need to leave [school] early and the worker can chat and have a check in with the parent beforehand.

Some staff members also noted a positive impact on engagement and participation rates when delivering digital interventions and described how service users were canceling sessions less frequently. Thus, the reach of digital services was also highlighted as beneficial for enhancing the uptake of services:

We had good attendance with [manualised programme] considering we thought Zoom might be more of a barrier for some parents.

A parent could still make an appointment even if they had forgotten, when you called them and they realised, they didn’t need to try get to the centre last minute or back up to the house, they would just take the call, also as a worker you can offer to accommodate or rearrange sessions more easily now too.

Staff members also highlighted that digital services could remove stigma as a barrier to engagement, as service users could attend from home rather than in person at service centers. Instances where digital social care services enhanced service user comfort were also described, particularly among separated parents and young people. Digital services were identified as being particularly well suited to teenage service users, in part because of their perceived familiarity and comfort with technology. Participating service providers also shared case examples of teenagers having their pets with them on a video call or painting while they participated in web-based sessions. These regulating activities were perceived as helping the young person feel safe during service delivery and facilitating a greater sense of control over their participation and engagement with social care services.

#### Perceived Disadvantages for Service Users

The perceived disadvantages of digital social care for service users are centered largely on access and the capacity to use technologies. Lack of digital literacy among service users has been identified as a significant challenge. Older service users, such as grandparent carers who lacked experience in using internet-based devices or smart devices, were identified as frequently lacking experience and confidence in using digital platforms. Other staff members also shared examples of parents who initially appeared disengaged from services, but it later transpired that they needed more guidance and support in engaging with digital technologies. Staff members also highlighted some instances where service users were uncomfortable working in web-based spaces due to self-consciousness and, consequently, did not enjoy seeing themselves on the screen. Web-based “fatigue” was seen as a barrier to engagement, particularly for school-going children who were engaging in remote education and parents who were working remotely from home during school closures and lockdowns.

Inadequate home spaces and access to technology and Wi-Fi were also identified as key barriers for service users. Although focus group participants described how most service users had internet access via their mobile phones, they often did not have Wi-Fi or a computer, laptop, or tablet. Staff members were also conscious that although checking in and connecting through video calls was very manageable from a phone, it was not ideal for attending formal programs or groups:

For groups like baby massage or a parent and baby group or any of the parenting groups it really isn’t ideal when parents join by phone, you can’t see them and baby properly, they don’t get to see who else is there, it’s doable but it’s a very small screen.

In addition, practitioners also described how families frequently only had access to 1 device with internet at home, which during school closures was needed to allow children to engage in distance learning, and this, in turn, reduced parents’ ability to engage in web-based services. Reliable connectivity was also identified as an issue, particularly outside of urban areas.

### Practitioners’ Challenges in Working With Families on the Web

#### Overview

Three subthemes were identified in relation to the challenges for Barnardos social care practitioners working in digital contexts: (1) developing and sustaining therapeutic relationships on the web; (2) privacy, confidentiality, and safeguarding; and (3) assessing the suitability of digital service delivery (Table 1).

**Table 1:** Overview of practitioner challenges in delivering digital social care interventions.

Subtheme	Illustrative quotes
Developing and sustaining relationships on the web	“It’s very daunting to meet for the first time on a Zoom call. Relationship building takes longer. If even the initial visit happens on the doorstep, it helps to put a face to the name.” “With new parents it can be difficult online, it’s hard to gauge their baseline across a screen and go through all the formalities, it’s hard to get a sense, particularly if only in contact once a week.” “When parents get upset, if you’re not in the same room with them, it’s really hard, you really feel for them. It can be hard to express empathy across a screen...kind gestures are lost, making them a tea, giving a tissue things like that.” “Sometimes when you want to show empathy in practice, you might be really still to show they have your attention and that your listening and you can hold what they are sharing, but with online you need to try nod more as it can be confusing for the service user and they might think they’ve lost you or your frozen.” “At the beginning of lockdown it took time to get familiar with the different apps and technologies that are available but once I figured it out, what works well, I found that I love online, I’m surprised at how much I do.” “All of the team have upskilled on what they give across on screen—there is a whole set of etiquette that has been developed. Even things like nodding when agreeing rather than saying “Yeah” as that can knock out the sound.”
Privacy, confidentiality, and safeguarding	“When you’re doing a child interview online and you want to speak about serious concerns in the home, the parent is around so you’re not getting a full picture and they may be coached into what they are saying or influenced.” “If you want to speak to a mam but dad could be in the house/another room or arrive as you’re chatting, and you might not be getting a full picture of what is happening. This is something that definitely was a worry and did not work online.” “When you walk into a home, you’re making observations with all of your senses, what you smell, what you see, what you feel, is the floor sticky? All of this gives you a greater sense of what is actually going on, how things are going, whereas you can’t get that in the same way online. It is easier for a family to say everything is fine.” “When you’re making calls or carrying out your work in the project, there are plenty of ears around, the parent could be in another room or your team generally witness your work, hearing what you said or how you speak—but at home doing your one-to-ones...It’s worth thinking about safeguarding practices for staff.”
Assessing the suitability of web-based service delivery	“You have to be really organised because you do not have the tools, your bag of tricks such as art supplies to call on. [When delivering in person] a young child might get distracted but you can pull from something and bring them back in.” “With one particular five year old it was a real challenge, the child was more interested in pressing the buttons on the screen than engaging in the activities.” “We normally might not have been able to accommodate this family but the flexibility and elimination of the barriers of travelling into the project meant that this family received the service they needed in a way that suited them [...] The child was also able to benefit from individual work online, whereas that mother would have never been able to get him down here with her work.” “For those parents who are working from home, they are able to fit sessions in around their breaks and working day. If they are running late or behind by 10 minutes to an online session, that’s fine, I’m not at the front door already waiting.” “Some parents engaged better online or the telephone as they felt less threatened or more in control of the session.” “Online work can create nervousness [...] particularly in relation to a consciousness of how they look, what they say, their home environment and feeling exposed. [...] children and young people attending our services can struggle with anxiety or have big things to talk about, the issues of increased anxiety and privacy need to be considered.”

#### Developing and Sustaining Relationships on the Web

The challenge of developing and maintaining therapeutic relationships was a central issue for the staff members involved in focus groups. Pivoting to digital social practice during the pandemic was experienced as easiest when a positive relationship with service users was already established. Overall, practitioners perceived communication via the web with new service users to be more challenging and, consequently, that it was more difficult to build up knowledge of new service user needs and to respond appropriately and sensitively.

During focus groups practitioners also commented that, when possible, initial contact and introductions with service users should take place in person. Disruption to existing relationships, however, was also highlighted by some participants, particularly those who worked directly with children. These participants felt that connection was harder to maintain through digital social care practice and that the absence of informal conversations and “small talk” that naturally occur had undermined the sense of connection. Challenges in holding difficult conversations with service users via the web were also described, particularly where child welfare concerns existed. For example, 1 participant described how a parent became disengaged following a child welfare referral and felt that “if those tough conversations had have happened face-to-face, I would have been better able to keep them connected.”

Similarly, other participants described the challenges of dealing with sensitive matters in a web-based space and adequately supporting service users when they became upset. For example, participants described how the skills, techniques, and gestures they usually used to help a service user regulate in these moments were impractical or unsuitable for web-based platforms. For example, routine practices such as creating “therapeutic silence” or gestures such as offering tissues had to be replaced with different behaviors to convey empathy and support for service users. Participants also described an urge to “fill the gaps” when talking with services users via the web and, consequently, felt they were “over talking.”

Despite these challenges, participants also reflected that as their familiarity with web-based delivery grew, they began to gain new skills that enabled them to communicate more effectively when delivering digital social care interventions.

#### Privacy, Confidentiality, and Safeguarding

Concerns about ensuring confidentiality and safeguarding were highlighted in the focus group findings. When working on the web with parents, both individually and in group-based programs, participants described being keenly aware that children were often also present at home. They also expressed concerns that where parents did not have access to a private, confidential space in their home, children may be able to hear sensitive conversations from which they should be protected. Similarly, fears that children could not always speak freely when engaging from home because of parents or carers listening to their conversations with practitioners during digital service delivery were also expressed.

Working with parents who may be experiencing domestic violence and abuse was also a significant concern, and in such instances, practitioners described how they needed to be extremely careful about what they said, in case anything could be heard by an abusive partner. In turn, this had a negative impact on their work.

Ensuring confidentiality of service users was identified as an issue when delivering group-based programs. Participating practitioners described instances of intrusions where third parties or other household members could be seen walking around or present in the room and voiced their concerns regarding who may be listening to conversations and discussions between group members. They also pointed out that service users might not be aware of these potential threats to confidentiality in group-based settings. Engaging in child protection assessments through digital platforms was viewed as highly challenging, as the ability to observe and gain a true sense of what was happening for the family was limited.

Occasionally, practitioners also highlighted personal privacy concerns. Some were anxious about engaging in service delivery from their own homes, where there was an absence of easily accessible support from colleagues. This led to staff members feeling more vulnerable as a consequence.

#### Assessing the Suitability of Digital Service Delivery

The importance of assessing the appropriateness of digital social care was emphasized across focus groups. Developmental considerations and service user ability to engage in digital services were identified as key factors. For example, older children and teenagers were frequently identified as adapting well to web-based service delivery. However, web-based service delivery with younger children, particularly those aged <8 years, was perceived as impractical because of communication barriers and lack of familiarity with technology. Maintaining the attention of younger children during web-based sessions was identified as a major challenge. Consequently, these sessions were experienced as particularly tiring.

Overall, assessing the suitability of working on the web on a case-by-case basis was emphasized as an important but nuanced process. Practitioners described experiences of working with high-needs families who adjusted well to digital service delivery and demonstrated significant engagement with interventions despite complex needs. Consequently, practitioners stated that it “really depended what was happening there and then especially for families that were in crisis.” Indeed, some benefits for previously “hard-to-engage” parents, working parents, or highly anxious service users were described. Nevertheless, it was also noted that for some service users with low self-esteem and confidence, digital interventions may give rise to increased feelings of self-consciousness and anxiety.

### Practitioners’ Personal Challenges and Training Needs

The lack of confidence among practitioners in their ability to work on the web was a consistent theme across focus groups. Insufficient access to appropriate equipment or Wi-Fi to adequately offer digital interventions was identified as an exacerbating factor. For instance, some described not having access to smart devices or work phones, which limited their ability or platforms available to engage parents with service users.

Staff members also described the experience of delivering via the web as “more intense” and tiring when compared with face-to-face service delivery. Although the time efficiency of web-based services was acknowledged, participants noted that because of reduced travel requirements, they were scheduling and attending more meetings and sessions than before. Consequently, social care workers said that “there is way more squeezed into a day” and that it was more difficult to “switch off” from work.

Diminished and reduced interaction with colleagues during this period was a challenge for practitioners, and focus group participants described how they missed the connection and collaboration with other staff.

Overall, there was a general perception among participating practitioners that digital service delivery would form an ongoing aspect of their practice. The participants also described how they would like to improve their capacity to deliver digital social care interventions. More specifically, participants felt that they would like additional support in using videoconferencing platforms more creatively. Focus group participants also described their experience of pivoting to web-based delivery as involving a “trial and error” process and felt that they would like to be more confident and knowledgeable in using more advanced features of these digital platforms. Indeed, some participating practitioners continued to experience a fear of technology and felt that they lacked sufficient literacy in the use of digital platforms, stating that they would like additional practical tips and advice about working on the web.

A need for “how to” resources that could be passed on to service users to support their engagement with digital social care interventions was also identified. Understanding and ensuring web-based safety for both service users and practitioners was also identified as an important area for capacity building. Developing greater knowledge and awareness in this area was identified as helpful in facilitating enhanced comfort in digital service delivery. Participants also felt that such training would enable them to offer better guidance and advice to service users regarding their engagement in digital support and interventions (Textbox 2).

**Illustrative quotes for practitioners’ personal challenges and training needs.**

- “Without the drive home or a de-brief in the project with the other worker while packing up, it took a while to decompress. Sometimes its heavy enough topics discussed in a session and then you’re supposed to just switch off and go downstairs to your own family, it was difficult.”
- “When you’re traveling in between meetings you have that time to unpack and process afterwards, that’s gone now.”
- “You can fit more meetings into your week now that they are online but online can make you too available in some ways, booking meetings now back to back means there is little time to reflect or process in between.”
- “...in terms of digital tools I would have stumbled across them rather than be equipped from the off...”
- “I think any training in working online will be beneficial to us and also refresh our ideas and ways we work online...”
- “I’d love to be able to click into a folder, go onto “creative activities for working with teenagers online” and within that be able to get ideas for a Quiz or an age appropriate activity or ideas that you can do across Zoom.”
- “It would be great to be able to use those really creative presentation apps [...] to become a really engaging facilitator online instead of just knowing enough to get by.”

## Discussion

### Principal Findings

The findings shed light on social care practitioners’ experiences with digital child and family social care services during restrictions associated with the COVID-19 pandemic. Overall, it should be noted that both benefits and challenges within the delivery of digital social care support were identified. Given the sudden and unprecedented changes required in social care practice because of pandemic-related restrictions, it is not surprising that mixed findings are evident [34].

There is a pressing need for social care services to expand their reach and engage with service users through digital means [35]. Digital social care offers a significant opportunity for a stretched and stressed system to meet increased demand [36]. However, it also creates several challenges including practitioner uncertainty and capacity gaps, digital inequality, and ensuring digital safeguarding protection [24]. Therefore, it is vital to reflect on and learn from the experiences of practitioners during the pandemic to inform future practice development.

Ensuring benefits and overcoming the disadvantages of digital social care require judicious decision-making in relation to how and with whom technological interventions are delivered. The findings highlight the potential of digital social care services to offer increased flexibility to service users, thereby improving access and reducing stigma. On the basis of the findings of previous studies [23,37], digital social care during the pandemic was seen as potentially enhancing participation among many service users who may otherwise disengage from intervention. Although young people were viewed as being well suited to digital interventions, practitioners’ experiences also revealed that service users with complex needs adapted well to, and benefited from, digital support. However, practitioner expectations and potential bias regarding the ability of service users to successfully engage in digital interventions may influence whether these technological solutions are made available [5,11]. Thus, ensuring a balance between the views and preferences of service users and practitioners is important.

The findings outlined here also shed light on the challenges for practitioners when working on the web, particularly fostering and maintaining therapeutic alliance with service users. Digital social care practice requires significant changes in the manner in which practitioners engage with service users and deliver support to children, young people, and their families [38]. Previous research has shown that practitioners are more likely to report lower levels of satisfaction with communication and relationships when therapeutic services are delivered digitally [39]. This propensity may be driven by practitioners’ history of training and experience of building therapeutic rapport in person rather than on the web [14].

Gaps in practitioners’ capacity for digital practices have long been recognized [40]. Capacity building is required to equip practitioners with new soft skills amenable to digital service delivery. For example, developing communication techniques that can translate as empathetic and supportive via screens may help practitioners feel more confident in engaging digitally with service users [41].

Our findings also indicate that service providers found that digital service delivery resulted in increased workload and reduced opportunities to decompress following intense service delivery [42]. Traditionally, team-based support has been important for social care practitioners dealing with the emotional challenges of working with vulnerable families [43]. Exploring new ways in which practitioners can interact with colleagues via digital platforms to facilitate access to emotional support and reduce the risks of burnout and vicarious traumatization may help promote practitioner well-being.

Practitioners’ experiences outlined here also revealed the ethical challenges posed by digital technologies. Notably, social care practitioners felt that where there were child protection or domestic violence concerns, digital social care service delivery reduced opportunities for “unfiltered” observation of child safety. Currently, there is limited literature exploring the effectiveness of social care and welfare assessments in the digital sphere. Further research on this issue is needed to track how digital innovations in social care practice continue to evolve and to identify how to best meet the needs and priorities of vulnerable children and families [36]. In a recent study, Ferguson et al [41] described how technology could be woven into a hybrid practice consisting of a range of complementary digital and face-to-face approaches. Therefore, it is important to remember that digital technology should not be seen as separate from in-person practice but rather as a flexible tool that equips practitioners with alternative spheres for relationship building and connection [42].

Overall, it is vital to develop an appropriate framework for ethical digital practices in social care systems [24]. Guidance for practitioners and appropriate technology is required to ensure that clients’ rights and privacy are supported. However, the findings outlined here emphasize that continuing inequities in digital skills as well as access to information technology and Wi-Fi connection are significant barriers to digital social care practice. The lack of appropriate spaces to engage in web-based support, gaps in digital literacy, and inadequate access to technology were identified as factors that may undermine the reach and effectiveness of digital social care practice. For service users, the development of “how to” resources that are tailored to needs of children and families may be beneficial in facilitating enhanced comfort and confidentiality during digital interactions and interventions.

However, it should also be noted that access to digital technology was also experienced by staff members. A recent social care review in the United Kingdom highlighted that practitioners are often working with underresourced and “clunky information technology systems” [44,45]. To be able to capitalize on any opportunity afforded by digital technology, addressing the digital divide and investing in information technological infrastructure and equipment within social care systems are required.

### Study Limitations and Strengths

This study focuses on the experience within 1 organizational context. The research was also conducted by staff internal to the organization, which may have led to “insider bias.” Steps taken to minimize any potential loss of objectivity included the collection of anonymized survey data, whereas reflexivity was also built into the qualitative analysis process to support the appropriate interpretation of the findings.

Despite these limitations, staff members had experience of delivering multiple digital services across a range of web-based platforms and with a range of different children and families, facilitating insight into the challenges and barriers of working remotely during the COVID-19 pandemic. Quantitative and qualitative data sources were used to strengthen the findings. Finally, this research was conducted during a period of considerable upheaval in the delivery of social care support. The delivery of digital interventions in social care remains underresearched; however, it is likely that this type of support will be used to a much greater extent following the COVID-19 pandemic. Thus, these findings provide unique and timely learnings for the field of social care practice.

### Conclusions

Recent research carried out during the COVID-19 restrictions, including this study, highlights how the shift to digital social care practice resulted in significant disruption. The benefits and challenges of digital services in social care systems must be recognized. It is likely that web-based service delivery and hybrid practices combining digital technology and face-to-face intervention will form an aspect of social care practice in the future. The findings reported here highlight the importance of developing professional knowledge and guidelines that can support practitioners in assessing when digital service delivery is appropriate and beneficial to service users. Procedures that can ensure safety via digital service delivery are also very important. Addressing the digital divide and adequate investment in digital technology for social care systems is a fundamental necessity before digital social care intervention can become routine practice. Overall, further exploration of how, when, and with whom digital technology interventions can be safely and ethically used in social care practice is needed. This research should deepen our understanding of how best to optimize the implementation and effectiveness of digital interventions in combination with human support.

## Data Availability

The data sets generated during this study are not publicly available because of lack of participant consent but are available from the corresponding author upon reasonable request.
